# Pretreating mesenchymal stem cells with electrical stimulation causes sustained long-lasting pro-osteogenic effects

**DOI:** 10.7717/peerj.4959

**Published:** 2018-06-11

**Authors:** Maria Eischen-Loges, Karla M.C. Oliveira, Mit B. Bhavsar, John H. Barker, Liudmila Leppik

**Affiliations:** Frankfurt Initiative for Regenerative Medicine, Johann Wolfgang Goethe Universität Frankfurt am Main, Frankfurt am Main, Hessen, Germany

**Keywords:** Bone marrow-derived mesenchymal stem cells, Direct current electrical stimulation, Osteogenic differentiation, Bone tissue engineering

## Abstract

**Background:**

Electrical stimulation (ES) has a long history of successful use in the clinical treatment of refractory, non-healing bone fractures and has recently been proposed as an adjunct to bone tissue-engineering treatments to optimize their therapeutic potential. This idea emerged from ES’s demonstrated positive effects on stem cell migration, proliferation, differentiation and adherence to scaffolds, all cell behaviors recognized to be advantageous in Bone Tissue Engineering (BTE). In previous *in vitro* experiments we demonstrated that direct current ES, administered daily, accelerates Mesenchymal Stem Cell (MSC) osteogenic differentiation. In the present study, we sought to define the optimal ES regimen for maximizing this pro-osteogenic effect.

**Methods:**

Rat bone marrow-derived MSC were exposed to 100 mV/mm, 1 hr/day for three, seven, and 14 days, then osteogenic differentiation was assessed at Day 14 of culture by measuring collagen production, calcium deposition, alkaline phosphatase activity and osteogenic marker gene expression.

**Results:**

We found that exposing MSC to ES for three days had minimal effect, while seven and 14 days resulted in increased osteogenic differentiation, as indicated by significant increases in collagen and calcium deposits, and expression of osteogenic marker genes *Col1a1*, *Osteopontin*, *Osterix* and *Calmodulin*. We also found that cells treated with ES for seven days, maintained this pro-osteogenic activity long (for at least seven days) after discontinuing ES exposure.

**Discussion:**

This study showed that while three days of ES is insufficient to solicit pro-osteogenic effects, seven and 14 days significantly increases osteogenic differentiation. Importantly, we found that cells treated with ES for only seven days, maintained this pro-osteogenic activity long after discontinuing ES exposure. This sustained positive osteogenic effect is likely due to the enhanced expression of *RunX2* and *Calmodulin* we observed. This prolonged positive osteogenic effect, long after discontinuing ES treatment, if incorporated into BTE treatment protocols, could potentially improve outcomes and in doing so help BTE achieve its full therapeutic potential.

## Introduction

The growing field of Bone Tissue Engineering (BTE), with its encouraging early outcomes, holds great promise as an alternative to conventional bone autograft treatments. BTE treatments, like autografts, use osteogenic stem cells, space occupying scaffolds, and signaling growth factors. However, BTE has the added advantage that these cells, scaffolds, and growth factors can be manipulated prior to treatment to maximize bone healing potential. Cell-based treatments have been shown to improve bone healing by acting primarily in the early stages of repair (Kon et al., 2000; Wan et al., 2007; Yamada et al., 2012), during active recruitment of skeletal stem cells. In the case of BTE, autologous mesenchymal stem cells (MSC) are the cell of choice, based on their relatively high osteogenic activity, isolation and expansion efficiency, and safety. With an eye towards optimizing the therapeutic effectiveness of BTE treatments, researchers have manipulated MSC to maximize their osteogenic potential. Examples of different approaches that have been tried include: pre-differentiation of MSC into an osteogenic lineage by stimulants like Fibroblastic Growth Factors (FGF-2) (reviewed in Mauney, Volloch & Kaplan, 2005), delivering genes via genetic techniques (reviewed in Qin, Guan & Zhang, 2014), mechanical strain (reviewed in Lavrentieva et al., 2013), and exposing MSC to electrical stimulation (Balint, Cassidy & Cartmell, 2013). These approaches, when used in *in vivo* models, have been shown to accelerate osteogenesis by delivering more mature cells into the defect making them capable of immediate bone formation, resulting in overall improved healing (reviewed in Mauney, Volloch & Kaplan, 2005).

The use of electricity to treat bone fractures is not new, having been used successfully in clinical settings since the 1970 s (Mollon et al., 2008). The efficacy of electrical stimulation (ES) as a method to enhance bone healing has been demonstrated in a number of pre-clinical and clinical studies (Connolly et al., 1974; Brighton et al., 1985); however, the concept of combining ES and BTE to improve BTE outcomes is new. Jing et al. (2016) showed that pulsed electromagnetic fields improve osteogenesis *in vitro*, and then *in vivo*, in a rabbit bone defect model; they demonstrated that it enhanced osteointegration into porous titanium implants (Jing et al., 2016). In *in vitro* studies we and others have shown that daily application of ES stimulates bone cell behaviours like proliferation, migration, differentiation, and adherence to scaffolds (Mobini, Leppik & Barker, 2016; Mobini et al., 2017). In these experiments bone marrow derived- (BM-MSC) and adipose derived-MSC (AT-MSC) were exposed to direct current ES causing an increase in osteogenic differentiation (Mobini et al., 2017; Hammerick et al., 2010). In subsequent *in vivo* studies, we exposed BTE treated rat femur defects to continuous ES and demonstrated enhanced bone healing (Leppik et al., 2018). From these studies it is clear that ES has a strong positive osteogenic effect on cells *in vitro* and this effect is directly transferrable to an *in vivo* BTE treatment. While these studies have demonstrated a clear positive osteogenic effect, what is not clear is the optimal regimen for delivering ES.

The aim of this study was to identify the optimal ES regimen needed to stimulate a positive osteogenic effect in MSC. To achieve this, we conducted a series of *in vitro* experiments in which we exposed MSC to ES for different amounts of time over a period of 14 days and measured the resulting effect on osteogenic differentiation.

## Materals & Methods

**Figure 1:**
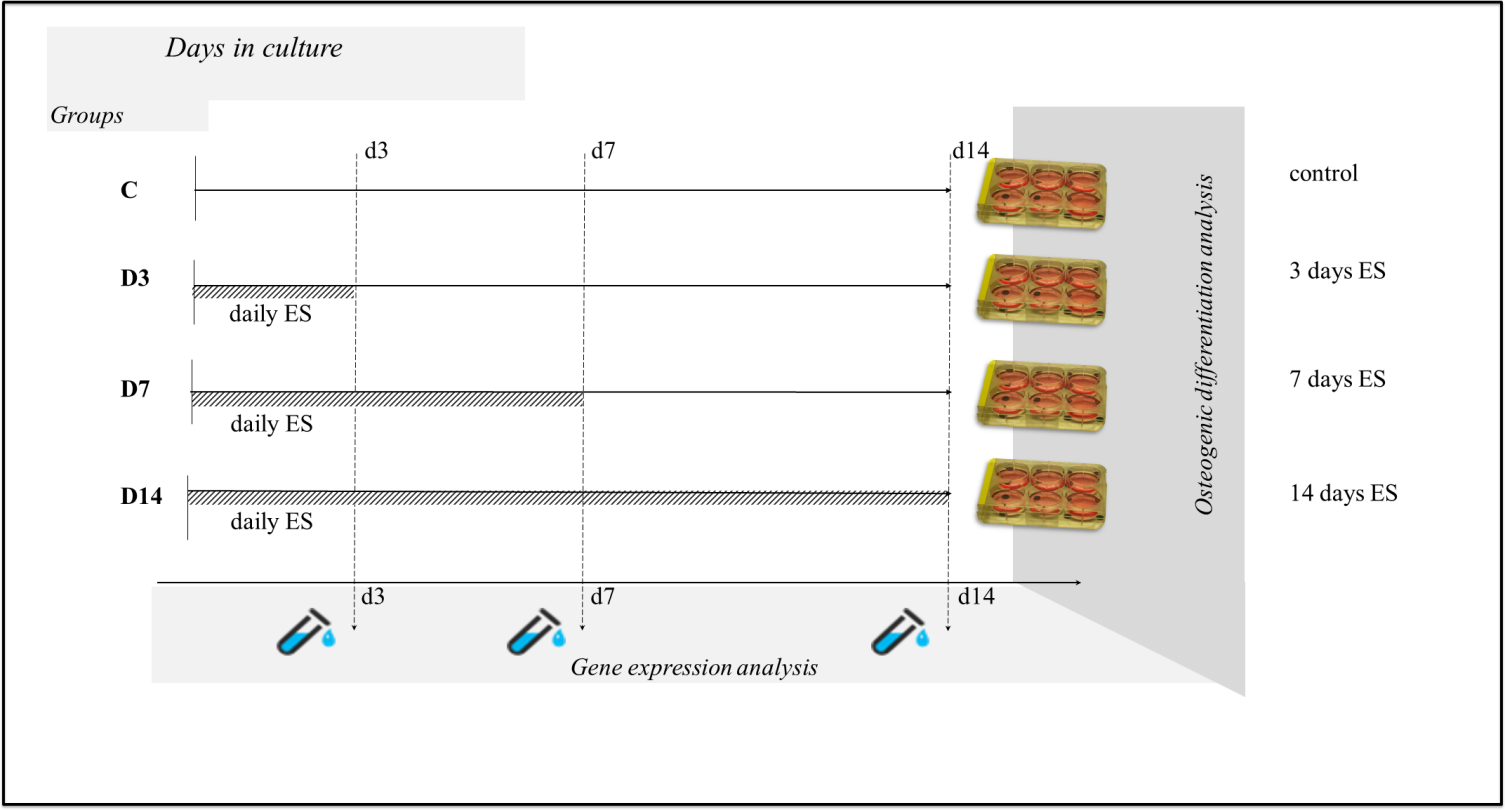
Experimental design. MSC were allocated into four groups: C (contol)- cells were treated the same as in the other groups but were not exposed to ES; D3- cells were exposed to ES for three days; D7- cells were exposed to ES for seven days; D14- cells were exposed to ES for 14 days. At Day 14 of culture osteogenic differentiation analysis was performed on all groups and gene expression analysis was performed at Days 3, 7, and 14 of culture.

To determine the ideal ES regimen for achieving increased MSC osteogenic differentiation we cultured MSC in osteogenic-supplemented medium for 14 days exposing them to 100 mV/mm for 1 h/day of direct current ES for the first three days (Group D3); for the first seven days (Group D7); and for all 14 days (Group D14), and then measured collagen content, calcium deposits, alkaline phosphatase activity and gene expression of osteogenic markers to assess osteogenic differentiation (Fig. 1).

### Cell culture in osteogenic medium

In order to avoid donor variation of bone marrow derived MSCs (BM-MSC), commercially available rat BM-MSC isolated from syngeneic Sprague-Dawley (SD) rats (RASMX-01001, Cyagen, CA, USA) were used in the study. Each cell stock vial was previously tested and certified by the manufacturer via surface marker expression and lineage differentiation testing. BM-MSC were thawed and cultured as described in detail elsewhere (Mobini et al., 2017). To assess osteogenic differentiation, 9 ×10^4^ cells (passage 5–6) were seeded into 6-well plates (TPP, Trasadingen, Switzerland) and after 24 h of culture (Day 0) growth medium was exchanged with osteogenic medium (OM) supplemented with 10^−7^ M dexamethasone, 10 mM β-glycerophosphate, and 0.05 mM ascorbic acid-2-phosphate (Sigma-Aldrich, Germany). Medium was changed every two to three days.

### Electrical stimulation of MSC

Forty eight hours after seeding, cells were exposed to 100 mV/mm for 1 h/day of ES for three, seven, or 14 days using a custom-made cell culture ES device (Table 1, group D3, D7, D14). A detailed description of the ES device and methodology is provided in Mobini, Leppik & Barker (2016) [12]. Control cells (no ES) were treated the same as cells in the other groups but did not receive ES.

**Table 1:** Experimental setup.

Group	ES duration	Analysis	Analysis	Analysis
		Day 3	Day 7	Day 14
Control (C)	-none-	Cell number, MTT, Gene expression	Cell number, MTT, Gene expression	Cell number, MTT, ALP, Collagen and Calcium deposits, Gene expression
Experimental D3	3 days			
Experimental D7	7 days			
Experimental D14	14 days			

### Measurement of cell metabolic activity

To assess whether exposure to ES was cytotoxic, cell metabolic activity was measured using MTT assay. Measurements were performed at Days 3, 7, and 14 of culture in electrically stimulated groups (Cell proliferation Kit I MTT, Roche, CH) and data was normalized to cell number obtained from a PicoGreen assay. The effect of electrical stimulation on cell number was evaluated using PicoGreen assay according to the manufacture’s protocol (Quant-iT™ PicoGreen^®^, ThermoFisher, Germany) at Days 3, 7, and 14. Briefly, cells were washed two times with PBS, treated with lysis buffer (400 mM potassium phosphate buffer, 2% Triton X100, 10 mM EDTA, pH7.0), sonicated (45 KHz, 10 min, +4°C) and cell lysates were used for DNA content measurements. Solutions with known DNA concentration in the same lysis buffer were used for calibration curve development and assays normalization. Lysates of a known number of cells were used for calibration curve development and cell number calculation.

To assess the effect the three different ES exposure times had on MSC osteogenic differentiation, collagen production, calcium deposition, alkaline phosphatase activity and osteogenic marker gene expression were measured in all groups and compared.

### Collagen content analysis

At Day 14 of culture, cells were washed twice with DPBS and fixed overnight with methanol (Sigma Aldrich, Heidelberg, Germany). Fixed cells were washed with DPBS and stained with 0.1% Picrosirius Red solution (Sigma Aldrich, Heidelberg, Germany) for 1 h at room temperature. Stained cells were washed repeatedly with 0.1% acetic acid solution and imaged using bright-field and fluorescence microscopes (TexRed filter) (Eclipse Ti inverted; Nikon Instruments, Tokyo, Japan).

### Calcium deposit measurements

At 14 days of culture, cells were washed with DPBS and fixed with 4% paraformaldehyde (Sigma Aldrich, Germany) solution in DPBS for 15 mins. Fixed cells were incubated with 0.02% Alizarin Red solution (Sigma Aldrich, Germany), in the dark, for 30 mins at room temperature. Stained cells were rinsed repeatedly with deionized water and imaged with a light microscope (CKX53, cellSens Entry 1.9 Software; Olympus, Japan) at a magnification of 10 ×. Cell calcium mineralization was assessed by semi quantification of Alizarin Red stain by acetic acid extraction and neutralization with ammonium hydroxide followed by colorimetric detection at 405 nm (Infinite 200PRO; Tecan, München, Germany) as described previously (Gregory et al., 2004). Data was normalized to DNA content.

### Alkaline phosphatase activity measurements

Alkaline phosphatase (ALP) assay was performed on cell lysates prepared in the same way as for cell number counts, and ALP activity was measured according the manufacturer’s protocol (SensoLyte pNPP Alkaline Phosphatase Detection kit; Anaspec Inc, CA, USA) in all groups at Day 14. Absorbance was measured at 405 nm using an Infinite 200PRO plate reader (Tecan, München, Germany). Readings were compared against the calibration curve of p-nitrophenol standards and normalized to DNA content.

### Osteogenic gene expression marker measurements

In order to analyze the effect the three different ES exposure times had on osteogenic marker gene expression, total RNA from cells was isolated using Aurum Total RNA Mini kit (Bio-Rad, Germany) according to the manufacturer’s recommendations. The quality and quantity of RNA were measured using gel electrophoresis and an Infinite 200PRO NanoQuant device (Tecan, München, Germany). DNase-treated RNA samples were reverse transcribed using iScript Select cDNA Synthesis Kit (Bio-Rad, Germany) according to the manufacturer’s instructions. Quantitative real time polymerase chain reaction (qRT-PCR) was performed using cDNA equivalent of 5 ng RNA and the SsoAdvanced Universal SYBR Green Supermix (BioRad, Puchheim, Germany). All samples were amplified in duplicates with a CFX96 Touch Real Time PCR Detection System (BioRad, Puchheim, Germany) using rat gene specific primers (Sigma Aldrich, Munich, Germany) and Runt-related Transcription Factor 2 (*RunX2*) (Qiagen, Hilden, Germany) (Table 2). Ribosomal Protein P1 (Rplp1) and Tyrosine 3-monooxygenase/tryptophan 5-monooxygenase Activation Protein Zeta (Ywhaz) were used as reference genes (Curtis et al., 2010). A melting curve analysis was applied to ensure specificity of the PCR procedure. Amplification products were also analyzed by gel-electrophoresis.

**Table 2:** qRT-PCR primers sequences.

Gene	Forward primer	Reverse primer
*Calmodulin*	TTTGACAAGGATGGCAATGGCT	TGTTAGCTTTTCCCCGAGGT
*ColIa2*	TTCCCGGTGAATTCGGTCT	ACCTCGGATTCCAATAGGACCAG
*Osteopontin*	GATGAACAGTATCCCGATGCC	TCCAGCTGACTTGACTCATGG
*Osterix*	CTGGGAAAAGGAGGCACAAAG	GGGTGGGTAGTCATTGGCATAG
*Rplp1*	GCATCTACTCCGCCCTCATC	GCATCTACTCCGCCCTCATC
RunX2*	*	*
*Ywhaz*	GATGAAGCCATTGCTGAACTTG	GTCTCCTTGGGTATCCGATGTC

**Notes.**

*ColIa2*, collagen type I alpha 2 chain; *Rplp1*, Ribosomal protein P1; *Ywhaz*, tyrosine 3-monooxygenase/tryptophan 5-monooxygenase activation protein zeta; * *RunX2*, Runt-related transcription factor 2 purchased from Qiagen (330523; Qiagen, Hilden, Germany).

### Statistical analysis

All experiments were performed in triplicate. Biological means and standard deviation of at least three individual samples per time point were calculated. Statistical significance between control- and experimental group data was evaluated via Wilcoxon–Mann–Whitney-*U*-Test for two groups analysis and for comparisons among 3 and more groups, Kruskal–Wallis-Test with multiple Conover-Iman-Comparison (Bonferroni-Holm-correction) was applied on BiAS for WindowsTM version 11.0 software (http://www.bias-online.de). Data are presented as mean ± SD and significance level was set at *p* < 0.05. Relative quantification of messenger RNA (mRNA) levels of the target genes was analyzed using the comparative *C*_*T*_ (threshold cycle values) method (2^−Δ*Ct*^) (Livak & Schmittgen, 2001). The results are presented as relative expression, which is expression of gene of interest normalized to housekeeping genes expression. Standard deviation (SD) was calculated with the Δ*C*_*t*_ value of three biological replicates.

## Results

### Metabolic activity/viability

**Figure 2:**
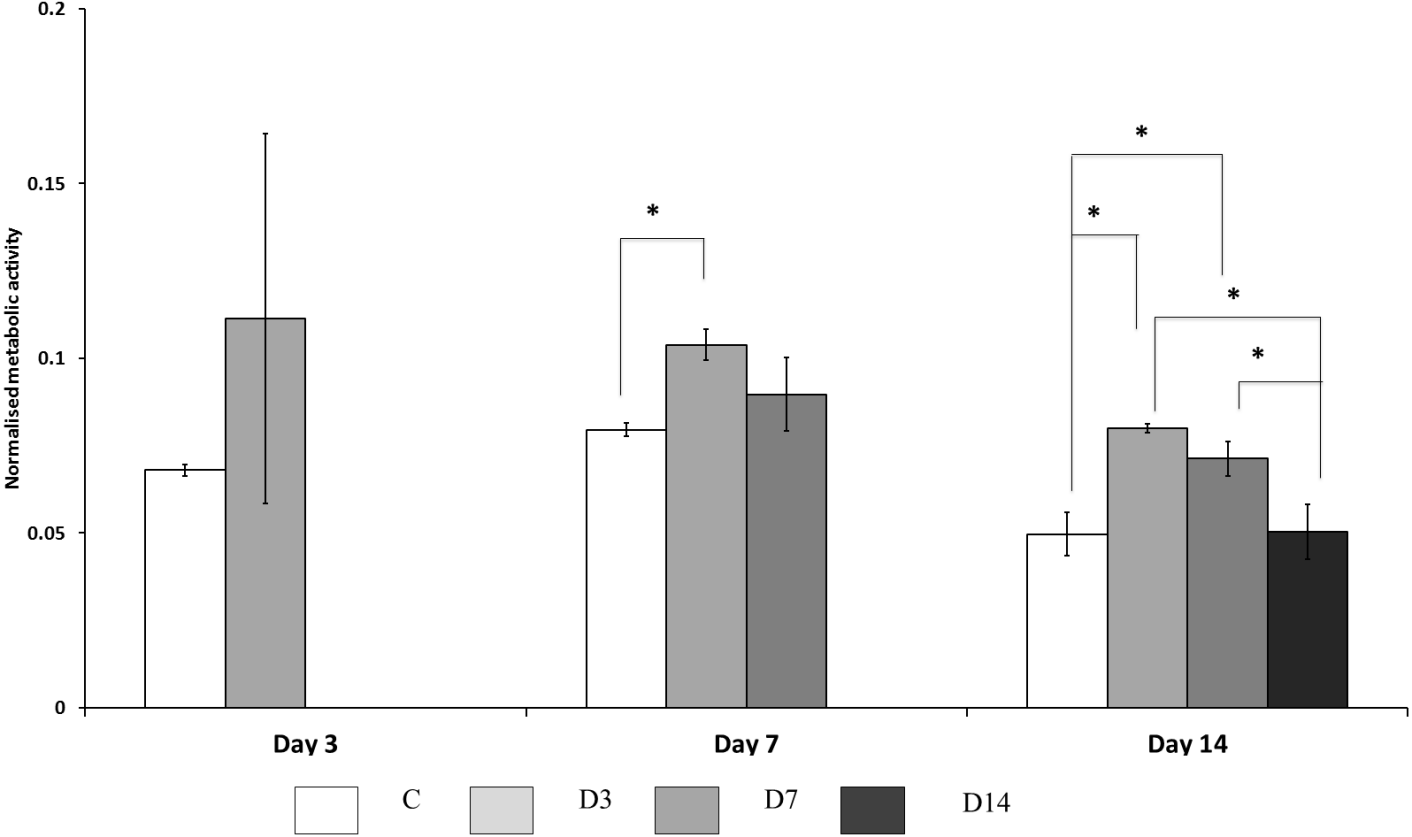
Cellular metabolic activity. Measured by MTT assay, comparisons made between ES, and non-ES (control) cells. Cells treated with ES for three days (D3) had significantly (*p* < 0.05) higher metabolic activity at Day 7 and 14 compared to control (C) cells. Cells treated with ES for three (D3) and seven (D7) days had significantly (*p* < 0.05) higher activity at Day 14 compared to control and D14 cells. Stars on bars indicate significant differences among groups.

The effect of three different ES exposure times had on MSC metabolic activity, and indirectly viability, was assessed using MTT assay (Fig. 2). At Day 3 of culture no significant difference was observed, however, electrically stimulated cells presented a tendency to increase cell activity by approximately 1.5 fold. At Day 7 of culture cells treated with ES for three days (D3 group) demonstrated higher activity than control cells. Cells from D7 group at this time point also showed propensity to more activity; however, this difference was not statistically significant. At Day 14 of culture, cells treated with ES for three and seven days (D3 and D7 groups) showed higher metabolic activity compared to control cells; while cells from D14 groups showed no difference in metabolic activity compared to control. Cells treated for 14 days with ES showed reduced metabolic activity compared to the other ES treated groups.

### Osteogenic differentiation

The effect of the three different ES exposure times on osteogenic differentiation was assessed by measuring collagen content, calcium deposition, and alkaline phosphatase activity at Day 14 of culture.

### Collagen content

**Figure 3:**
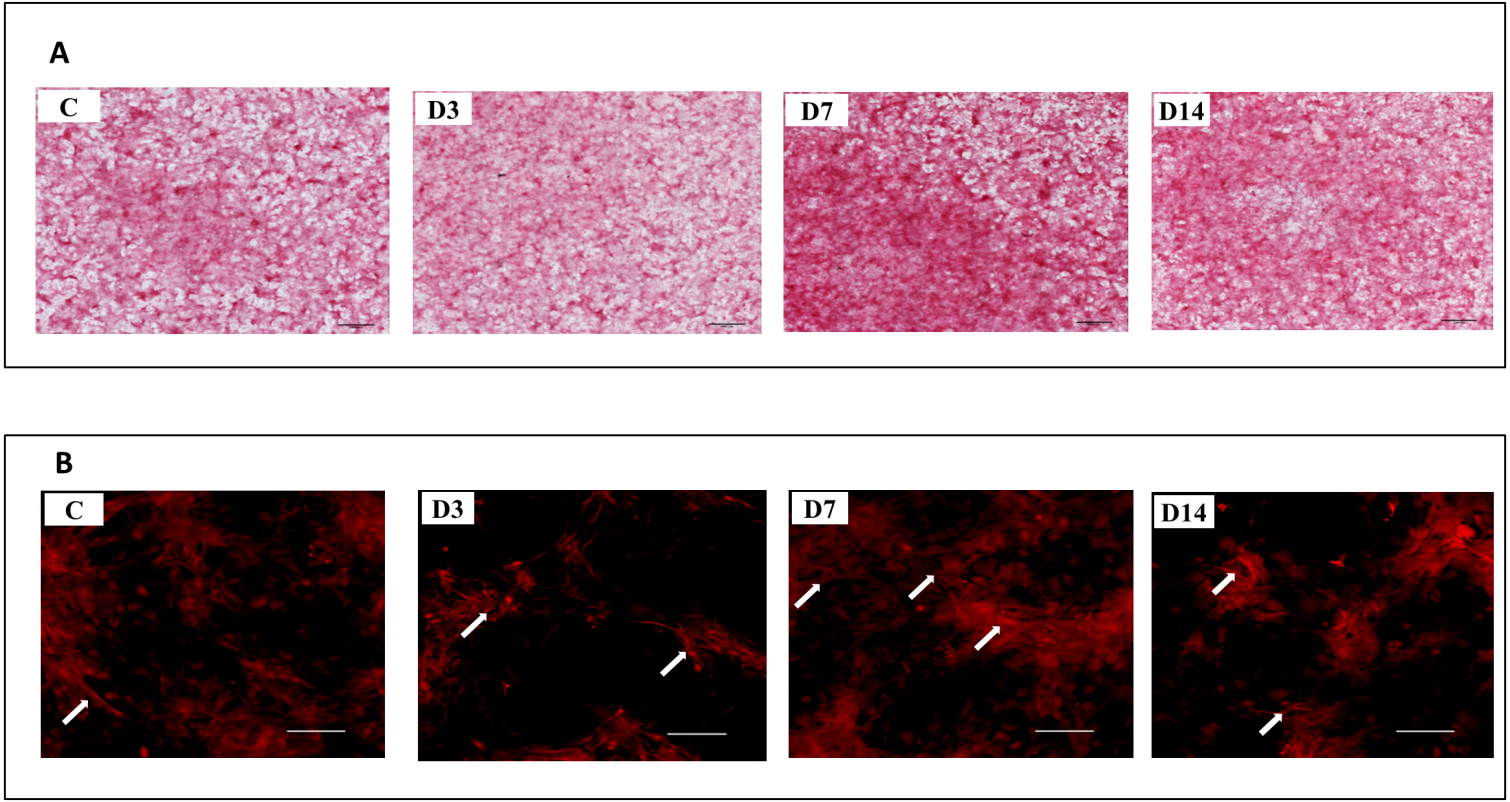
Representative images of collagen deposition at Day 14 of culture. (A) Representative images of control cells (C), and cells exposed to three (D3), seven (7D), and 14 (D14) days of ES stained with Sirius Red at Day 14 of culture. (B) Stained collagen showed by fluorescence microscopy. Arrows indicate collagen fibers highly condensed in D7 and D14 cells. A lower amount of fibers was observed in D3 and control cells.

All four groups (D3, D7, D14, and C) stained positive for collagen, however the intensity and distribution of staining varied (Fig. 3A). Morphological differences in collagen fibrils were observed between cells exposed to ES and those not exposed (controls) (Fig. 3B). Collagen fibers in ES treated cells were longer, densely packed and organized in complex networks. Higher amounts of collagen were observed in the D7 and D14 groups, compared to the D3 group.

### Calcium deposition

**Figure 4:**
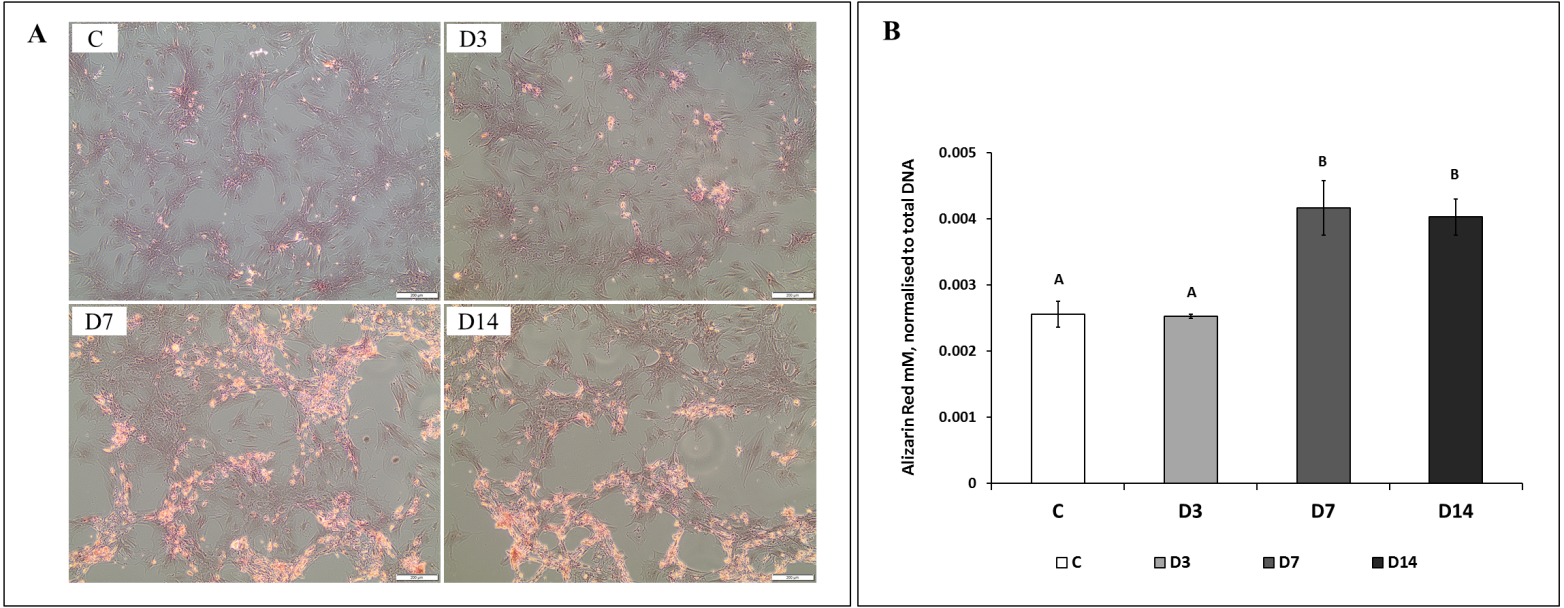
Representative images and semi-quantitative analysis of ECM mineralization at Day 14 of culture. (A) Representative images of cells not treated (C), and treated with ES for three (D3), seven (D7), and 14 (D14) days, stained with Alizarin Red at Day 14 of culture. (B) Quantification of Alizarin Red stained calcium deposits. Amount of calcium deposits in D7 and D14 cells was significantly (*p* < 0.05) higher in comparison to control (C) and D3 cells. No difference was detected between the amount of calcium in D7 and D14 cells, or between C and D3 cells.**** Different letters on bars indicate significant differences among groups.

The effect of the three different ES exposure times had on calcium deposition was measured and deposits were detected in all the groups (D3, D7, D14, and C) at Day 14 (Fig. 4A). Exposure to ES caused considerable increase in calcium deposition and cell morphology changes. Measurements of calcium deposition (Fig. 4B) showed significant differences between the groups, with D7 and D14 having the most, and D3 and C the least amount of deposits. No differences in the amount of calcium deposits were detected between groups D7 and D14, or groups D3 and C.

### Alkaline phosphatase activity

**Figure 5:**
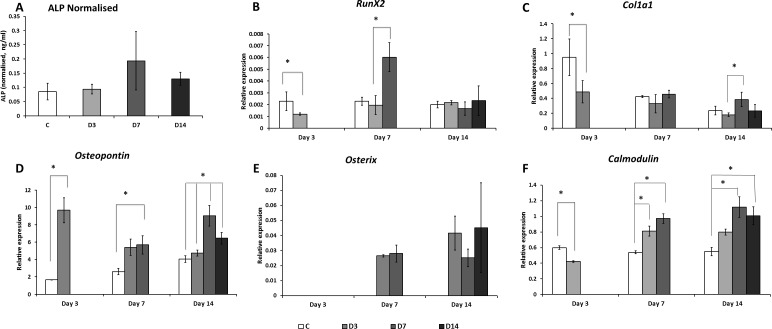
Alkaline phosphatase and osteogenic marker genes expression. (A) Alkaline Phosphatase (ALP) expression was measured in all groups at Day 14 of culture. No significant difference in ALP amounts was detected between the groups. (B) Expression of *RunX2*, (C) *ColIa1*, (D) *Osteopontin*, (E) *Osterix* and (F) *Calmodulin* genes were measured by mean of qRT-PCR at Day 3, 7, and 14 of culture. Asterisk indicates significant (*p* < 0.05) differences among groups within the same time point.

Despite no significant difference was found in ALP activity measured at 14 days among the groups (Fig. 5A), cells treated with ES for seven and 14 days (groups D7 and D14) revealed higher ALP activity compared to the control and D3 groups.

### Osteogenic marker gene expression

ES exposure had a significant effect on *RunX2*, *Osteopontin*, *Osterix* and *ColIa1* gene expression (Fig. 5). While at Day 3 of culture ES exposure decreased expression of the early osteogenic marker gene *RunX2*; at Day 7 of culture, ES applied for seven days caused a significant increase of *RunX2* expression (Fig. 5B). Expression of the osteogenic marker gene *Osterix* was detected in cells exposed to ES at Day 7 and 14 of culture however was not detected in control (Fig. 5E). The amount of these transcripts was similar in D3, D7, and D14 cells at both time points. Expression of the *Osteopontin* gene was highly increased in ES treated cells as early as three days of culture (5.8 fold) and persisted at Day 7 of culture compared to control (Fig. 5D). Different times of ES exposure had different effects on gene expression. Cells exposed to ES for only three days (D3) had increased *Osteopontin* gene expression at Days 3 and 14 of culture, but not at Day 7. Cells exposed to ES for seven days (D7) had increased expression of this gene at all three time points, with two peaks at Days 3 and 14 of culture. When measured at Day 14 of culture, cells exposed to 14 days of ES (D14) revealed lower amounts of *Osteopontin* transcripts compared to those exposed to seven days of ES (D7), yet these values were still higher than those found in controls and D3 groups. At Day 3 of culture, expression of *ColIa1* was lower in ES treated cells than in controls. At Day 7 of culture, no effect of ES on *ColIa1* gene expression was detected (Fig. 5C). On the other hand, expression of this marker was significantly higher at Day 14 of culture in cells treated with ES for seven days (D7), in comparison to cells treated for only three days (D3). Expression of the *Calmodulin* gene was significantly increased at Days 7 and 14 of culture in cells treated with ES for seven and 14 days, but not three days. At the earlier time point (Day 3), *Calmodulin* expression was decreased in ES-treated cells (Fig. 5F).

## Discussion

Although not fully understood, MSC osteogenic differentiation is a well-coordinated process regulated by complex signal transduction and transcriptional networks that include phases of proliferation, maturation, matrix synthesis and mineralization (reviewed in Heino & Hentunen, 2008). ES has been shown to increase intracellular calcium activity leading to significant cytoskeleton reorganization that cause cell movement (Yang et al., 2017). Wang et al. (2016) showed that 200 mV/mm of direct current ES for 4 h enhanced migration, proliferation and differentiation of rat BM-MSC. In our own studies, we demonstrated that 100 mV/mm of direct current ES administered for 1h/day, for 21 days, is capable to improve mineralization and expression of osteogenic marker genes (Mobini, Leppik & Barker, 2016; Mobini et al., 2017). In the present study to determine the effect of different ES exposure times on MSC osteogenic activity we exposed cells to 100 mV/mm of direct current ES for three, seven, and 14 days, then measured metabolic activity, organic matrix formation and mineralization, and expression of different osteogenic marker genes, at these different time points. We found that cells, exposed to ES for three days had the highest metabolic activity at seven and 14 days of culture. We also found that metabolic activity of cells treated for seven days does not differ from that of non-treated control cells at Day 7 of culture, and is higher at Day 14 of culture. Moreover cells treated with ES for seven and 14 days showed a tendency to decrease metabolic activity throughout the culture time. This could be an indication of cytotoxicity, resulting from prolonged ES exposure, but more likely it indicates increased differentiation, since it is known that metabolic activity decreases at late stages of differentiation (reviewed in Wanet et al. (2015) and Hu et al. (2016)).

Cells exposed to seven and 14 days of ES showed increased osteogenic differentiation at Day 14 of culture, as indicated by elevated levels of collagen and calcium deposits. As the main component of extracellular matrix in bone, collagen plays an important role in osteogenesis with altered collagen crosslinking having been associated with decreased osteogenic differentiation in MC3T3-E1 mouse cell line (Fernandes et al., 2009). In this study, we found that collagen fibrils and calcium deposits were more prominent in cells exposed to ES for seven and 14 days than those exposed for only three days and non-treated cells. These observations were supported by our gene expression data. The highest expression of the osteogenic marker genes; *ColIa1*, *Osteopontin* and *Calmodulin*, measured at Day 14, was in cells treated with ES for seven days. In cells continuously treated with ES for the entire 14 days, expression of *Osteopontin,* and *Calmodulin* genes was not as high as those exposed for seven days**,** but higher than in control cells. Cells treated with ES for three days showed only increased expression of *Osterix* and *Osteopontin* genes. No significant difference in ALP activity was detected in three, seven, and 14-Day ES-treated groups at day 14 of culture. ALP transcription and protein expression is known to take place at the early stages of osteogenesis, before cells reach the ECM mineralization stage (Aubin, 2001). At 14 days of culture we detected calcium deposits in all groups, suggesting that this time point might be too late for detecting differences in ALP activity. Overall these data show that seven days of ES treatment has the strongest effect on MSC osteogenic differentiation, whereas three days of ES exposure has minimal effect. These findings support a recent report by Zhu, et al. in which rat BM-MSC were stimulated with direct current ES during different stages of osteogenic differentiation, and ES exposure in the first seven days of culture (early stages of differentiation) was found to be the greatest (Zhu et al., 2017).

In order to understand why seven and 14, but not three days, of ES exposure enhances MSC osteogenic differentiation, we analyzed the change in expression patterns of osteogenic marker genes over time in ES treated, and non-treated (control) cells. We found that *Osterix* gene was up regulated in all ES treated groups, whereas the others only reacted to prolonged (seven and 14 days) ES treatment. In all ES-treated groups expression of *Osterix* gene was increased at Day 7 and 14 of culture, as compared to control cells. Transcription regulation of *Osterix* gene has not been well studied, but is thought to be controlled by a transcription network, signaling pathways and epigenetic regulation (Sinha & Zhou, 2013). *Osterix* gene is considered a direct target of *RunX2* regulation; however, its expression can be up-regulated in the absence of *RunX2* as well (Lee et al., 2003). Our data suggests that ES affects *Osterix* expression not through a *RunX2* pathway, as its expression was up regulated in the absence of enhanced *RunX2* expression.

We observed important differences in *RunX2* and *Osteopontin* gene expression patterns between cells exposed to three versus seven and 14 days. In cells treated with ES for three days there was no increase in *RunX2* at any time point of culture, while an increase in *Osteopontin* expression was detected at Days 3 and 14. This would indicate that three days of ES exposure is insufficient to increase *RunX2* expression, and thus increase osteogenesis. This was evidenced by the significantly lower collagen and calcium deposition we observed in these D3 cells compared to those exposed to seven and 14 days. *RunX2* expression regulation is complex and includes epigenetic mechanisms, such as miRNAs and several histone modifying enzymes (Huang et al., 2010; Di Yang et al., 2013; Rojas et al., 2015). Further experiments are necessary to determine why seven and not three days of ES is sufficient to enhance expression of *RunX2* genes. We found *Osteopontin* expression to be increased in all ES treated cells, however the expression patterns differed among the different ES-treatment groups. Whereas three days of ES exposure caused *Osteopontin* expression to peak at Day 3, it later decreased at Days 7 and 14 of culture. In cells exposed to ES for longer times (seven and 14 days), *Osteopontin* expression peaked a second time at Day 14. This second expression peak correlates with up regulated earlier *RunX2* and *Osterix* genes, both known to be key inducers and regulators of osteogenesis (Carroll & Ravid, 2013). Early up-regulation of *Osteopontin* gene, in the absence of *RunX2* and *Osterix* genes, observed in all ES-treated groups at Day 3 was unexpected. We speculate this could be a direct effect of ES on the transcription of this gene. The *Osteopontin* gene is reactive to H_2_O_2_ (Lyle et al., 2014), which is known to be one of ES’s faradic products. While faradic products are recognized to be cytotoxic in higher concentrations, small amounts have been shown to have positive effects on cells proliferation, migration and differentiation and are believed to be key players in ES-induced bone healing (Bodamyali et al., 1999; Rhee, 2006; Balint, Cassidy & Cartmell, 2013). While in our experimental setup we did not measure faradic products (Mobini, Leppik & Barker, 2016), in similar experiments others described the presence of small amounts of H_2_O_2_ (Wang et al., 2016) which could be responsible for the increase in the expression of *Osteopontin* genes we observed. Fujita et al. (2014) hypothesized that reactive oxygen species (ROS) induced dead cells could be retained in the culture and serve as nuclei for calcification. It is possible that the decrease in cell number and increase in calcification we observed in our D7 and D14 groups could have been caused by ES induced ROS molecules present in the culture medium.

The observed increased expression of the *Calmodulin* gene in cells treated with ES suggests the participation of the Calcium/*Calmodulin*-signaling pathway in ES treatment and correlation of this increase with duration of ES treatment. Zhang et al. (2016) showed that in AT-MSC, ES activates ion flux through calcium channels, which they attributed to the induction of different reaction cascades. Expression of osteogenic gene markers due to intracellular calcium oscillations has been demonstrated to depend on the time of electrical stimulus application (Liu et al., 2017). In this study, three days of ES treatment was not enough to induce an increase in *Calmodulin* expression and stimulate osteogenic differentiation, while seven and 14 days of ES treatment was efficient to increase *Calmodulin* expression.

Despite increasing numbers of publications focused on the effects of electrical stimulation on stem cells, the detailed mechanisms explaining how cells sense, interpret and transform signaling remains unclear. Recent studies highlight the importance of primary cellular cilium organelle in sensing and transducing electrical current in osteogenic response in adipose derived MSC (Cai et al., 2017). The first interaction between ES and cells is most likely to occur in the cell membrane, in which ion channels (Wen et al., 2012; Zhang et al., 2016) and surface receptors (Wolf-Goldberg et al., 2013) are proposed to be the biochemical signaling mechanisms. Activation of calcium channels by ES could trigger downstream signaling pathways and activate components such as Ca^2+^/Calmodulin dependent kinase II (Catterall, 2011). It was also shown that redistribution of membrane integrins could activate the MAP kinase signal transduction pathway (Miranti & Brugge, 2002), which is known to play an important role in osteogenic differentiation (Binétruy et al., 2007). Mechanotransduction, reactive oxygen species (ROS) generation, and other mechanisms are also under investigation (reviewed in Sart, Song & Li, 2015). While identifying these mechanisms was beyond the scope of this study our findings suggest the possible involvement of Calcium/*Calmodulin* and ROS production mechanisms in MSCs reaction to direct current ES.

The increased expression of osteogenic gene markers we observed in cells treated for seven days with ES could be responsible for the induced osteogenic differentiation; also demonstrated by increased calcification and collagen levels. Remarkably, exposing MSC to 100 mV/mm of ES for 1 h/day for seven days, and then stopping ES exposure, the positive osteogenic effects persisted long after ES treatment was discontinued. This finding suggests that ES may activate a switch that causes sustained, long-lasting positive osteogenic activity in these cells. This could represent a new ES pre-treatment approach to be translated into TE applications and achieve optimal healing.

## Conclusions

In conclusion, we showed that while three days of ES is insufficient to solicit positive osteogenic effects, seven and 14 days significantly increase MSC osteogenic differentiation. Importantly, we found that cells treated with ES for only seven days maintained this pro-osteogenic activity long after discontinuing ES exposure. The observed increase in *RunX2* expression and activation of Calcium/*Calmodulin* pathway caused by seven days of ES exposure, may initiate a cascade of reactions that are responsible for the enhanced osteogenic differentiation we saw after the ES stimulus was discontinued. Our findings demonstrate that exposing BM-MSC to ES early, in the first seven days of culture, significantly increases osteogenic differentiation, while additional exposure beyond this does not seem to provide additional benefit. In future studies, we will attempt to reproduce this persistent osteogenic effect *in vivo* by exposing MSC + scaffold to ES *ex vivo* then implant this pretreated construct into a large bone defect and see if the positive osteogenic effect translates into improved bone healing. If successful, this new approach could improve BTE outcomes, allowing BTE treatments to achieve their full potential.

##  Supplemental Information

10.7717/peerj.4959/supp-1 Data S1 Raw data RT-qPCR; MTT, ALP and PicoGreen measurements

## Additional Information and Declarations

### Data Availability

The following information was supplied regarding data availability: The raw data file is uploaded as a Supplemental File.
